# Healthcare delivery and information provision in bariatric surgery in Germany: qualitative interviews with bariatric surgeons

**DOI:** 10.1186/s12913-021-06629-4

**Published:** 2021-07-05

**Authors:** Jessica Breuing, Nadja Könsgen, Katharina Doni, Annika Lena Neuhaus, Dawid Pieper

**Affiliations:** Institute for Research in Operative Medicine (IFOM), Witten/Herdecke University, Witten, Germany

**Keywords:** Healthcare, Bariatric surgery, Obesity, Semi-structured telephone interview

## Abstract

**Background:**

There are several healthcare professionals involved in health information provision regarding bariatric surgery, such as bariatric surgeons, nutritionists, and medical doctors in outpatient settings. Trustworthy health information supports patients in understanding their diagnosis, treatment decisions, and possible prognosis. Therefore, it is necessary to provide health information on bariatric surgery. This study has two distinct objectives. The first is to outline the delivery of healthcare regarding bariatric surgery in Germany. The second is to describe the information provision within healthcare delivery.

**Methods:**

We conducted 15 semi-structured telephone interviews with bariatric surgeons between April 2018 and February 2019. The interviews were audio recorded and transcribed verbatim. The interview guide consisted of four sections (information about the clinic/surgeon and surgical procedures, preoperative procedure, postoperative procedure, information needs). The transcribed interviews were analyzed using qualitative content analysis supported by MAXQDA software.

**Results:**

The pre- and postoperative processes differed substantially between clinics. Additionally, every bariatric clinic had its own information provision concept. There were several cost-related issues the surgeons claimed to be relevant for patients, such as nutritional blood tests or postoperative psychotherapy. These issues were often caused by unclearness of responsibility within the medical disciplines involved.

**Conclusion:**

Healthcare delivery in bariatric surgery in Germany is heterogeneous in terms of pre- and postoperative care. Therefore, preoperative information provision between the clinics differs. The impact of this heterogeneous healthcare delivery and information provision on patients’ information needs regarding bariatric surgery should be further investigated among patients and other healthcare professionals involved.

**Supplementary Information:**

The online version contains supplementary material available at 10.1186/s12913-021-06629-4.

## Introduction

In 2016, more than 650 million adults worldwide (13%) were living with obesity [1]. Obesity unlike other diseases is preventable, but without any intervention, its reversal is uncommon. Compared to non-surgical interventions, such as lifestyle modifications or pharmacotherapy, bariatric surgery (BS) especially sleeve gastrectomy (with and without modifications) or Roux-en-Y-bypass [2] are most effective in the treatment of severe obesity. The number of bariatric surgeries is rising internationally, with the highest number of bariatric surgeries taking place in the USA/Canada with 154,276 bariatric surgeries in 2013 (44 procedures per 100,000 inhabitants) and Germany with a total of 7126 bariatric surgeries (8.8 procedures per 100,000 inhabitants) [3]. In 2020, there were 88 certified competence and reference clinics for bariatric surgery [4] in Germany. If BS is indicated (body mass index > 40 kg/m^2^ or > 35 kg/m^2^ with comorbidities [5]), costs for BS are normally reimbursed by (statutory) health insurance funds (HIFs) in Germany. For reimbursement, it must be shown that weight management program (including nutrition therapy, exercise, and behavioral therapy) have failed [6]. Therefore, patients must take weight management program prior to surgery over a period of 6 months.

There are several healthcare professionals involved in the process of BS, such as bariatric surgeons, nutritionists, and medical doctors in outpatient settings (general practitioners, endocrinologists, plastic surgeons), who are involved in health information provision. Trustworthy health information for patients is most likely given by healthcare professionals [7] and supports patients in understanding their diagnosis, treatment decisions, and possible prognosis [8]. Since health literacy is defined as “the degree to which individuals have the capacity to obtain, process, and understand basic health information and services needed to make appropriate health decisions” [9], providing health information is a step towards increasing patient’s health literacy. In addition, health literacy is one of many factors impacting weight loss after surgery, so high health literacy provides successful weight loss after BS [10]. Patients undergoing BS are at high risk of anemia due to vitamin B12, iron, or folic acid deficiency [11]. Mahawar et al. demonstrated only 45% adherence (complete compliance) to micronutrient supplements and that patient education was important for taking the supplements [12]. It is necessary to provide education, e.g., in the form of health information on BS. Additionally, access to dietary supplements and nutritional monitoring is important to prevent or identify any deficiencies.

The aim of this study was to describe the delivery of healthcare and explore the provision of information on BS in Germany from the perspective of bariatric surgeons.

## Methods

### Design

The study was approved by the Witten/Herdecke University Ethical Committee (224/2017). All methods performed in accordance with the Declaration of Helsinki. Written informed consent was obtained from all participants. There was no incentive for participation.

This qualitative interview study is part of a larger research project to identify the information needs of patients undergoing BS. We designed a project with three qualitative interview studies (surgeons, patients and nutritionists) to identify the information needs of patients undergoing BS and map the information provision within the pre- and postoperative hospital process. The present study is one of these studies, targeting the bariatric surgeons’ view on information needs and their description of information provision at their clinic as well as their perception or experience of patients undergoing bariatric surgery. We defined information provision as all processes involved in giving healthcare information to patients. This includes the form of information (personal, e.g., in one-on-one appointments or in groups, written, e.g., as a flyer or webpage), timing of the information provision (pre−/postoperative), and the information given. The other interview studies focused on nutritionists and patients’ views on information needs.

Choosing a qualitative approach was necessary because there is no standard in Germany on how to initiate the preoperative (information) process for patients. Therefore, the preoperative process, including information provision, had to be collected individually.

We used the Standards for Reporting Qualitative Research [13] to report our results.

### Recruitment

The current study is based on qualitative semi-structured interviews with bariatric surgeons who were sampled nationally in Germany. Eligible participants were bariatric surgeons working in a clinic for BS and willing to describe their clinical characteristics and their pre- and postoperative healthcare delivery. The audio-recorded telephone interviews were conducted by an experienced nutritionist (JB) with expertise in qualitative research and obesity/BS. The interviews were conducted at a time and location of the participant’s choice.

Eligible participants were identified through the German Society for General and Visceral Surgery. This society certifies competence and reference clinics for BS and provides a list of all certified clinics, including contact information [4]. We used this list to contact the clinics by e-mail and asked them to recommend one of their surgeons to participate in our study. Furthermore, we used snowball sampling to reach clinics/surgeons that were not on this list due to an ongoing re−/certification process. Since we contacted all clinics listed, there was no predefined sample size to stop recruiting further than the number of reminders (three per clinic/surgeon) sent per e-mail. Detailed information about the study (aim of the study, duration of the interview, publication), along with privacy statements, was given to each participant prior to the interview.

Overall, we contacted *n* = 68 clinics. Three clinics rejected instantly because of their heavy workload; *n* = 44 clinics did not respond at all. Seven clinics were interested at first but did not respond to further communication. The overall response rate of the clinics was 20.6% (14/68). The contact to the clinic/surgeon which conducted a pretest interview is not included in the response rate.

### Data collection

The data collection period was April 2018 to February 2019. The interview guide (Supplement 1: interview guide) was designed prior to the interviews and consisted of four main sections (general information about the clinic/surgeon and surgical procedures, preoperative procedure, postoperative procedure, information needs). It was reviewed, tested, and modified within a pretest interview with one experienced bariatric surgeon. All participants received a shortened version of the interview guide to prepare their given information on, e.g., the number of procedures each year.

The interviews started with demographic information of the surgeon and general information on the clinic. The second section started with open questions to map the content of the first appointment with a patient (patient-doctor appointments) and the information given in these appointments. The surgeons also stated all preoperative organizational requirements and standards a patient had to do, including weight management program (appointments with nutritionists/surgeons, group meetings, dietary/exercise programs). Furthermore, the surgeons were asked about the most common questions patients had prior to surgery. Afterwards, the surgeons had to describe the postoperative process at the clinic and list the most common problems and questions of postoperative patients. The last section focused on information provision, information needs, and future approaches for information provision.

### Data processing

The audio files of the semi-structured telephone interviews were transcribed verbatim by an external agency.

Based on the interview guide, data codes were developed predetermined to the interview analysis by one researcher (JB) and checked by another (NK). The data codes were divided into nine groups with several subgroups:

1. Participants characteristics, general data
2. Preoperative care
3. Preoperative information
4. Costs
5. Postoperative care/information
6. Postoperative problems
7. Information needs
8. General Problems
9. Solutions

Furthermore, rules of coding and code specifications were defined for each code (Supplement 2: data coding system).

### Data analysis

The transcribed interviews (including the pretest interview) were analyzed using qualitative content analysis [14] supported by MAXQDA software. Two researchers (JB and NK) independently analyzed one-third of the interviews with the predetermined data codes. After discussion and consensus, the data codes were modified again, and the given codes were adjusted. After achieving reasonable interrater reliability, further analysis including code modifications were conducted by one researcher (JB).

## Results

We conducted *n* = 15 semi-structured interviews. The duration ranged from 21 to 57 min with a mean time of 38 min. Nine surgeons were male (67%), and six were female (33%).

Characteristics of the clinics.

The clinics had a minimum of two and a maximum of five bariatric surgeons and performed 70 to 500 bariatric surgeries each year. The clinics prioritized performing either sleeve gastrectomy or gastric bypass (mostly Roux-en-Y bypass) or both procedures equally (clinic characteristics are listed in Supplement 3: Characteristics of the clinics).

### Preoperative

#### Preoperative delivery of healthcare

**Fig. 1:**
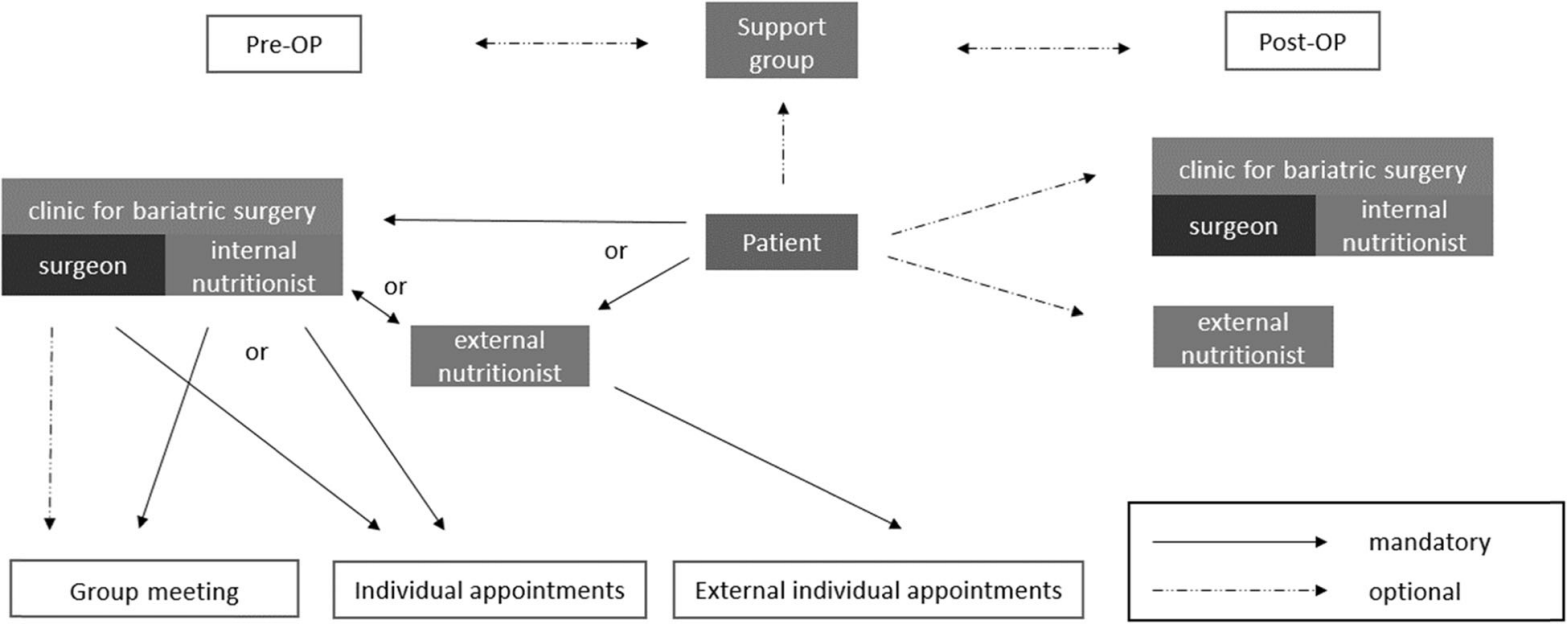
Possible ways of pre- and postoperative care for patients undergoing BS

There were several mandatory and optional ways to interact with all BS parties (clinic, surgeon, nutritionist, support group). All possible ways of pre- and postoperative care for patients undergoing BS are described in Fig. 1. First, there are two different preoperative starting points for patients: clinics for BS and nutritionists. It is mandatory for all patients to go to a clinic for BS and speak to a bariatric surgeon and a nutritionist based on weight management program prior to surgery in order to obtain cost coverage from their HIF. The appointments with the surgeon were individual patient-doctor conversations, while appointments with the nutritionist could be either individual (within the clinic or external) or in group meetings with other patients. If the nutritionist was external, e.g., in outpatient settings, there was little to no information sharing regarding each patient between the surgeon and the nutritionist. Consequently, neither of them was aware of the information already given to the patients by the other. Rarely, a surgeon joins the (nutritional) group meetings, which are often held by a nutritionist. One surgeon explained that they had different procedures for weight management program, nutritional blood tests, or psychotherapy for patients with one specific HIF and patients from other HIFs.

*B03: “We also do weight management program, but basically within the framework of our [Name of the HIF] program. It's difficult for patients from the other insurance companies because we can't cover the costs.”*

*B03: “For patients who are in the [Name of the HIF] program, the laboratory control is paid by the [Name of the HIF]. Patients with other insurance companies, there are a few insurance companies that cover the costs. And there are some health insurance companies that do not cover the costs at all, so you have to refer them to their general practitioners.”*

*B03: In the [Name of the HIF] program anyway, and in other programs we have to see that we connect them either with us in-house or with a cooperation partner, if there are problems, but then we try to do it more in-house, because then the wire is shorter, yes.*

The duration of the first appointments with the surgeon ranges from 15 to 60 min, while most appointments last 30–45 min. Surgeons met patients in one or two individual appointments.

Most clinics (*n* = 13) were directly cross-linked to a support group and often provided premises for support group meetings. These support groups were usually self-sufficient and organized by former patients. All surgeons who had knowledge of an existing support group said that they are for both pre- and postoperative patients. Sometimes there are separate groups for pre−/postoperative patients. Some surgeons visited the support group meetings periodically to answer questions. If asked, most of the surgeons outlined the important role of support groups while indicating that the information given by the support group is based on experience and not necessarily evidence.

*B10: “[Support Groups] Super important. Therefore, it’s like a safe haven. Some of them [patients] don’t have that many relations or some feel like they are failures. To find themselves in a group of other sufferers. (…) Therefore, it’s somehow very informative and partly very opinion-forming, too.”*

#### Preoperative information provision

##### Information provision approaches

Most clinics used individual appointments with the surgeon and one additional approach, and some used several approaches (e.g., flyer, homepages, social media). One surgeon stated that they only used individual appointments with the surgeon to inform their patients. Most clinics had written information, e.g., flyers or information on their homepages. Three surgeons noted that they used and delivered information material provided by a medical device manufacturer. Social media was often used to provide information to a broad public. A blog with general and special information, e.g., on alopecia or obstructive sleep apnea, was designed by one surgeon. Another surgeon had a Facebook page with further information. One surgeon outlined the amount of already existing information on the internet, so the clinic saw no need to create their own written/online information material.

Additionally, seven surgeons had informational group sessions with either a surgeon or a nutritionist. In one clinic, this group session was the only information approach, so the patient-doctor meeting had the aim of settling the surgical procedure and not giving further information. One surgeon stated that they used different information approaches depending on the health insurance company of the patients because one health insurance company had their own approach within this clinic.

##### Costs

In response to the question whether postoperative costs, such as costs for dietary supplements or plastic surgery, were mentioned preoperatively in the patient-to-doctor appointments, one surgeon expressed:

*B01: “Dietary supplements absolute right, but we’re talking about 50 cents each day. That’s not a high cost to take and plastic surgery- abdominoplasty is paid by the health insurance company.*

Another surgeon commented on plastic surgery costs:

*B04: “Abdominoplasty was performed normally after one year after stabilization of weight. This is mostly paid by the health insurance funds. The patients know that. Other things, such as sagging breasts or loose upper arms, are partly for self-paying patients. This you have to tell patients.”*

Most surgeons declared that they informed patients about costs for supplementation, and approximately half of the surgeons provided additional information about the cost of plastic surgery or gave that information on demand, the other half did not comment on this.

##### Preoperative frequently asked questions (FAQ)

All but one surgeon stated that there are many questions before the surgery even though they indicated that many patients are well informed about the different procedures. There seem to be three categories of questions. First, there are general questions about the different procedures, pros, and cons as well as the risks of each procedure, weight loss, supplementation, and complications, regardless of the amount of information already given to the patients in that matter. Second, there are specific questions about medical issues such as medication use, the chance of becoming pregnant after surgery, or present comorbidities. Last, there are questions regarding everyday life, such as duration of sick leave, ability to work out after surgery, or potential plastic surgery.

### Postoperative

#### Postoperative delivery of healthcare

Postoperative care mostly included 1–2 appointments with the surgeon. Some surgeons gave a timeframe of 15–20 min, and some said that the duration depends on the patient’s issues. Usually, patients present 1–3 times in the first year after the procedure and annually thereafter.

##### Organizational problems

There were some problems mentioned by the surgeons. Severe problems were postoperative nutritional blood tests, pre- but more importantly postoperative psychotherapy and cooperation with general practitioners. All three problems had economic reasons in common.

The responsibility of postoperative nutritional blood tests is uncertain to patients and partly to clinics/surgeons. Surgeons tried to find solutions to provide the screenings and outline the importance of these screenings.

B13: “*Then, there is the question regarding vitamin controls [nutritional blood tests], the vitamin controls done by the general practitioner. Some general practitioners refused. In addition, they [the patients] then ask us, where to get these vitamin controls and if they are necessary. The answer is yes, and we offer vitamin control within postoperative care. This does not cost anything for the patients, this is billed via the university outpatient department.”*

B6: *In my opinion, an annual blood test should be done. Not only a blood test but also a vitamin check in the lab. We tried some sort of an arrangement in thirds. One-third [of the costs] is taken by the endocrinologist; if the patients were treated there before, one-third is taken by the general practitioner, and one-third is taken by us.*

*B15: “We also see that in the day-to-day practice because it isn’t settled yet who should bear the costs for this laboratory controls. This is extremely different from state to state.”*

Postoperative psychotherapy is not offered in one of the clinics, mostly because of the lack of financial support by health insurance funds. However, some clinics cooperated with internal or external psychotherapists in some form of on-demand offer.

We asked surgeons their opinions on how many patients should use postoperative psychotherapy:

B03: “*Well, at least half of them, maybe more. Well speaking from experience, their psychological problems did not manifest within the first two years.”*

Another surgeon announced that they were about to set up a psychotherapy consultation hour. The surgeon was further asked why they set up these consultation hours:

B09: “*It was rather on demand of the patients, but we saw a need for this. We tried to set these consultation hours for upcoming special questions. Therefore, patients know who to call. (…) However, this will not cover the request of all patients. Somehow it would be reasonable if a large proportion of patients receive additional behavioral therapy in some form. We tried to cover this by conversations with our nutritionist or even with medical doctors. However, in the end, we are not psychotherapists. We can’t provide this in a way a psychotherapist could through decent counseling.”*

#### Postoperative information provision

##### Information provision approaches

There were no postoperative information provision approaches other than the individual appointments with the surgeons. However, some clinics were linked to support groups that could be joined after surgery.

##### Postoperative FAQ

**Fig. 2:**
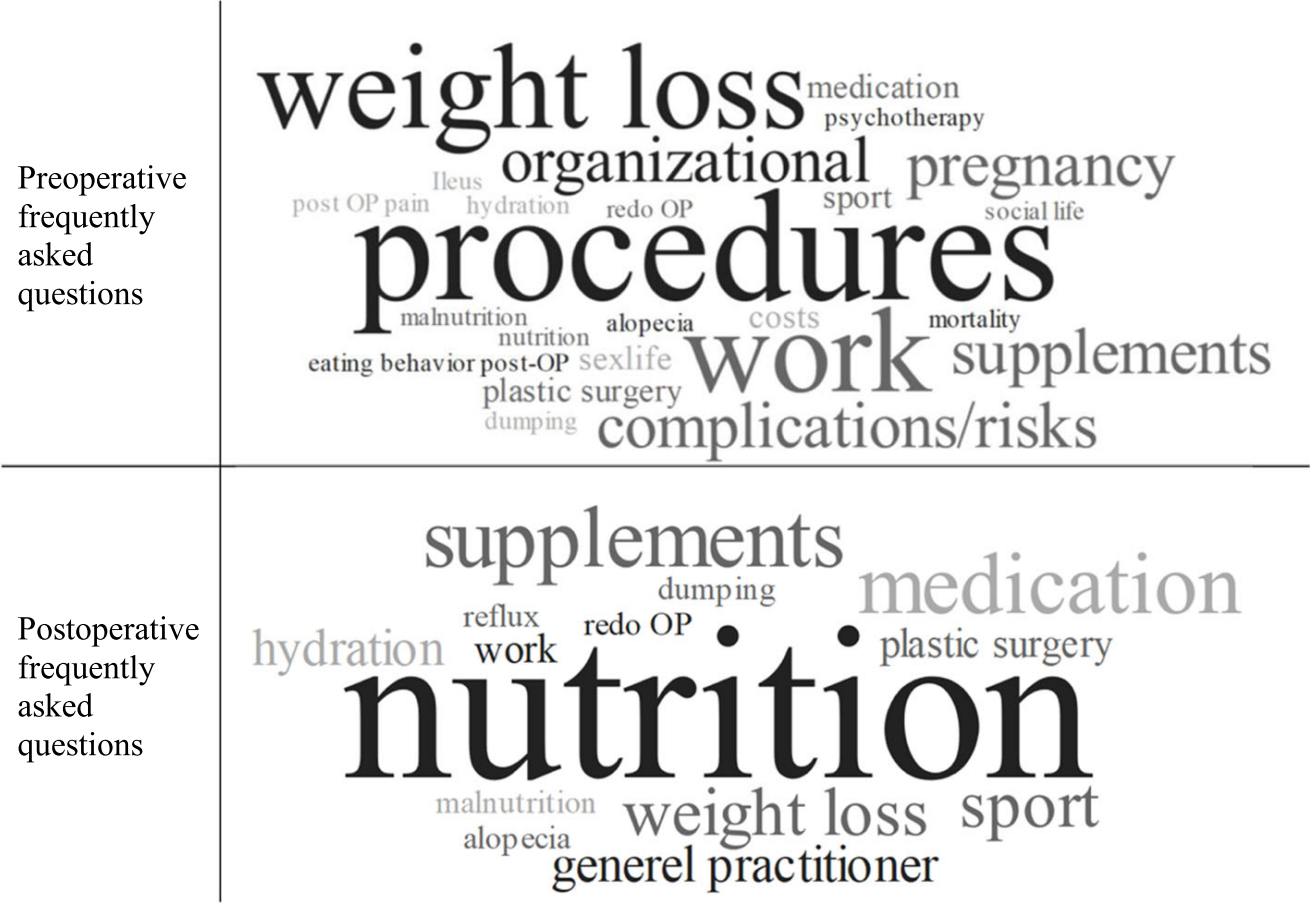
Word cloud of the frequently asked questions pre- and postoperative: visual comparison of frequency of all used subcodes

Other than the preoperative FAQs, the postoperative FAQs are more precise and include fewer issues (see Fig. 2). Postoperative questions related directly to the new life situation and adapting to these. Most questions centered on nutritional problems, medication use, or weight loss, especially in comparison to other bariatric patients.

##### Challenge in information provision

Finally, the surgeons were asked to express their opinion of the greatest challenge in information provision in the context of BS. There were three categories of given answers. First, the internet and its ambivalent influence on the given information.

*B06: “Well, the biggest challenge is the internet. They [the patients] are well informed, but with totally unselected information. And that’s the biggest problem - in my opinion. They had a bunch of information, with unknown state of accuracy. We sometimes incorrectly postulate thinks and inversely they do know more than we thought they knew. That’s difficult. That you don’t always know the source of the patients’ information”.*

Second, there seems to be an overload of information. The dosage and timing of the information given was stated by one surgeon as a key element in information provision.

*B05: “To don’t overload them [the patients]. They are filled up with information. You must consider carefully, when and which information. Don’t only think that more is more”.*

Third, there is the individuality of each patient regarding their level of education, compliance, health literacy, or even personality, which requires individual, customized information provision.

*B08: “The individuality. Well, to customize depending on the patient’s needs. To their level of intelligence, on their understanding of this medical issue, on their compliance. Does he [the patient] even care about such things? Well, I think, this is the biggest challenge.”*

## Discussion

### Healthcare and information provision

The healthcare processes regarding BS in Germany are not standardized. We found heterogeneous healthcare processes within the 15 clinics reviewed. Each clinic had its own proceedings regarding the preoperative procedure (e.g., number of appointments with the surgeon and/or nutritionist, presence of group sessions), information provision, and postoperative care. Conversely, a patient receives different intensities of care and information approaches depending on the choice of clinic and sometimes even depending on the HIF. To name only one of these factors, the duration of the individual appointment with the surgeons ranges from 15 to 60 min. Therefore, one surgeon only spends 15 min to provide all information, while another other one needs 60 min. There could be different reasons for this, one may be a different intensity of given information. Some clinics used group sessions to inform patients, while others informed patients one-on-one. The dynamics of group sessions could intimidate rather shy or restrained patients. Some surgeons refer to webpages that deliver information on BS. However, web-based information often differs enormously in quality [15, 16].

Support groups are useful to patients to share common experiences and often use social media such as Facebook to communicate [17]. Especially in postoperative settings, patients seem to require support groups to struggle with weight loss or regain [18]; therefore, support groups predict long-term weight loss [19]. Bariatric surgeons outlined in the interviews that the information given within the support groups is more likely based on experience and not necessarily evidence. Perhaps support groups could benefit from inviting a healthcare professional on a regular basis to help them gather and provide health information, as one bariatric surgeon already did. After BS, there are fewer and shorter appointments with the bariatric surgeon. To provide effective postoperative weight loss, an alternative could be an eHealth strategy [20, 21].

Surgeons stated that most patients are well informed even though they had some questions pre/postoperative. Comparing the preoperative FAQs to the postoperative FAQs, there seemed to be fewer questions postoperative, but the questions postoperative were more specific. While some patients had been informed prior to surgery about special aspects, e.g., weight loss after surgery, these patients still asked the same questions regarding weight loss after the surgery. Therefore, even if the patients had been given information, they could have unsatisfactory and unmet information needs. A need for additional information seemed to be common [22]. Therefore, other aspects of information provision could be causing the additional need for information, which should be investigated further. However, information provided by healthcare professionals should be equally ensured to each patient.

### Cost-related problems

Regardless of the clinic, there seems to be a major lack of postoperative care through unsettled questions on costs and responsibility. Due to different health insurance funds as well as differences within the federal states it is not always obvious who is entitled to carry out the tests in order to be paid for them. Some clinics performed postoperative nutritional blood tests, and some referred to the general practitioner or the endocrinologist. The German guideline on BS [5] stated that periodic nutritional blood tests should be performed twice within the first year post-OP and annually after the first postoperative year. There is no statement on who should perform this nutritional blood tests.

Some statements given in the interviews demonstrate various problems in cost-related information provision. First, the amount of costs for supplementation was underestimated by one surgeon *(“(…) we’re talking about 50 cents each day*”). There are various providers of dietary supplements in different price ranges in Germany. Regardless of the economic status of the patient, these rising expenses are worth knowing. On further enquiries, the surgeon admitted that they did not inform patients about the cost of supplementation, but the surgeon wanted to supply this information in the future. The need for further information on dietary supplements is already described in adolescent BS patients [23].

Regarding plastic surgery, some cost-related issues are indirectly linked to the patient’s HIF. In Germany (statutory) health insurance is mandatory. Plastic surgery after BS deals with excess skin and could improve weight control [24] but can also be challenging [25]. Most HIFs refund abdominoplasty but not plastic surgery of excess skin of the extremities. Therefore, information on possible plastic surgery should be more present and not only on demand in preoperative information provision. The information should be precise regarding the different plastic surgery procedures and the costs covered by HIF.

In the absence of psychiatric disorders, pre- or postoperative psychotherapy in the context of BS is not covered by most statutory HIFs because obesity is not defined as a psychiatric disorder but as a somatic illness [6]. Some of the clinics tried to provide on-demand psychotherapy support, but mostly only for patients in acute need. Neither pre- nor postoperative psychotherapy is offered as standard care in the context of BS by German HIFs. Nevertheless, treatment of preoperatively manifested psychological/psychiatric diseases is reimbursed by the HIFs. Psychosocial problems [26] as well as increasing suicide risks for patients [27] after BS are well documented. However, preoperative psychological interventions such as counseling or cognitive behavioral therapy neither improved postoperative treatment adherence for lifestyle changes [28] nor weight loss [29]. Still, postoperative intervention should be designed, implemented, and paid by HIFs to first screen, detect, and support psychosocial/psychiatric problems and then offer support for adherence to lifestyle changes and weight loss.

Healthcare delivery in BS in Germany is heterogeneous in terms of pre- and postoperative care. Therefore, preoperative information provision differs. The impact of this heterogeneous healthcare delivery and information provision on patients’ information needs for BS should be further investigated among patients and other healthcare professionals involved in the healthcare delivery of BS.

## Limitations

A limitation of this study is the small sample size. Although full saturation cannot be claimed we managed to explore the provision of information on BS in Germany from different clinics and could describe different problems. Additionally, we only interviewed surgeons who nominated themselves to participate, which may lead to a bias. Furthermore, we only focused on bariatric surgeons´ view on information provision and information needs. To include patients´ view, we conducted interviews with patients who have undergone bariatric surgery. The results of this study will be published soon and will add another important view.

## Supplementary Information

**Additional file 1.**

**Additional file 2.**

**Additional file 3.**

## Data Availability

The datasets generated and analyzed during the current study are not publicly available because of ethical concerns regarding privacy and confidentiality of participants. Datasets, however, are available from the corresponding author on reasonable request.

## Abbreviations

BS: Bariatric surgery
FAQ: Frequently asked questions
HIF: Health insurance fund
