# Supplementary material for: Healthcare delivery and information provision in bariatric surgery in Germany: qualitative interviews with bariatric surgeons
Source: BMC Health Serv Res. 2021 Jul 5;21:659. doi: 10.1186/s12913-021-06629-4 (PMC8258934; doi:10.1186/s12913-021-06629-4)
Supplement: Supplementary file 1 — Additional file 1. [file 12913_2021_6629_MOESM1_ESM.pdf]

The original German version of the interview guide has been translated with DeepL (https://www.deepl.com/translator).

Interview guide - bariatric surgeons

Introduction

Hello my name is Jessica Breuing from the Institute for Research in Operative Medicine at the Witten/Herdecke University in Cologne. We have been in contact before. I am very pleased that you support our study " Information needs of bariatric surgery patients" by participating in this interview.

You know from our last e-mail and the shortened interview guidelines sent out in advance what our interview will be about. Do you have any questions before we start?

It is important to emphasize that there are no right or wrong answers, and that you just freely answer honestly. We want to use the interview to determine the information processes around bariatric surgery, not to evaluate you as a physician.

Do you agree that the interview will be recorded via recording device? This will help us with the subsequent transcription and evaluation.

In any case, your data will be used exclusively for this current project and only in pseudonymized form. In other words, the interview results will be published without being linked to your name. Do you agree?

Okay, let’s start. (Switch on the recording device!)

I would like to start by asking a few parameters that specifically represent the bariatric procedures performed.

- How many procedures per year are performed at your facility?
- How many bariatric surgeons do you have in your [name of clinic/department]?
- Which procedures are performed at/at the [name of clinic/center]?
    - Which procedure is performed most often? [If applicable, can you justify why this procedure is performed most often]?
- Does your department/clinic cooperate with one or more nutritionists?

  IF YES

    - (If not already mentioned) How many nutritionists do you collaborate with?
    - Are they self-employed or employed by the hospital?
    - Is there an exchange between you as surgeon and the nutritionists?
    - Do patients see the nutritionist before <u>and</u> after surgery?

Pre-OP

In the following, I would like to outline the process of preoperative preparation.

Would you please give me a short description of an initial consultation with the patient?

- (If not already mentioned)
    - Do you work with the “multimodal therapy concept”?
    - How long does such an initial interview take on average?
    - What information is given in the initial consultation?
    - Does the initial consultation equals an medical briefing?
    - At what point is the surgical procedure determined?
        - What is the patient's role in the decision-making process?

Does the initial consultation equals an medical briefing about the different surgical procedures, their risks as well as consequences (change of diet, possible excess skin, etc.)?

I would like to know what specific content is given by the attending surgeon as part of the medical briefing. For example, what information is given about the risks of the various operations, consequences for nutrition and everyday life, medications and costs?

- In what form does the patient receive this information? (written, verbal, reference to literature or other sources).
- (If not already mentioned)
    - What information regarding the different surgical procedures does the patient receive?
    - What concrete consequences for the patient's diet and daily life are explained?
    - What information regarding nutritional supplements and their costs does the patient receive?
    - (In relation to the nutritionist) For which information do you refer to the nutritionist?

Surely, patients still have many questions after the explanation within the medical breifing. What questions are frequently asked by the patient before the operation?

- Why do you think [frequently asked questions] occur?
    - If you had to categorize the most frequent questions, would they tend to be questions to deepen information that has already been given, issues that have not yet been addressed, or emotionally driven questions? Would you say there is a category of questions that patients ask particularly frequently?
    - this issue addressed at any point?

I would like to expand on your answer to the category of frequently asked questions by the patient.

- What would you say are the most frequent questions asked to expand on information that has already been given?
- What are the most frequent questions asked about issues that have not yet been addressed?
- What are the most frequent motivations for questions that relate to fears? (Fear of complications, fear of aesthetic changes (e.g. excess skin, scars), fear of dietary changes, etc.).
    - Which questions arise most often in this context?

Post-OP

Now that you have described the preoperative procedure in detail, I would like to talk about the postoperative medical care of the procedures performed.

Could you please describe the general post-op procedure? Post-op means the entire period after the operation, including the time after discharge.

(If not already mentioned)

- What is the average length of a follow-up consultation?
- How often does the patient present after surgery? At what intervals does the patient present for follow-up?
- What questions are frequently asked by the patient after surgery (during the time the patient is no longer in the hospital)?
- What are the most common problems patients report after surgery?
    - Would you say that the problems raised by the patient are procedure-specific e.g. dumping or more general e.g. problems with diet or life change?
    - Take the problem [most common problem mentioned].
        - What do you think causes this problem?
        - Would there be a way to prevent this problem?
        - Is there information that would have minimized the problem for the patient or made the problem easier?

Now I would like to ask you a few final questions, which mainly relate to your personal assessment of information provision.

What do you see as the biggest challenge in providing information to patients in bariatric context?

Do you already have solutions in mind for [name challenge]?

Can you think of tools that could simplify information transfer on both sides?

We have now reached the end of the interview. Thank you very much for your participation and for your time.
