# Supplementary material for: Healthcare delivery and information provision in bariatric surgery in Germany: qualitative interviews with bariatric surgeons
Source: BMC Health Serv Res. 2021 Jul 5;21:659. doi: 10.1186/s12913-021-06629-4 (PMC8258934; doi:10.1186/s12913-021-06629-4)
Supplement: Supplementary file 2 — Additional file 2. [file 12913_2021_6629_MOESM2_ESM.pdf]

Supplement 2: Data coding system

Code Groups	Codes	Rules of coding	Code specification
participants characteristics, general data	Gender	not applicable	Separate extraction
	Number of bariatric surgeries each year	numbers	
	surgical procedures	context	
	most frequent surgical procedure	context	
	Number of bariatric surgeons at the clinic	numbers	
	Cooperation with nutritionists	context	y/n und internal/external
	Number of nutritionists at the clinic	numbers	
	nutrition counseling pre/post/both	context	
	exchange of expertise between surgeons and nutritionists	context	Obesity board etc.
	Conservative therapy y/n	context	If they are offering conservative therapy alone instead of surgery
Pre-OP care	- Groups sessions y/n<br>- Support group y/n	context	
	- Time interval between application by the insurance company and surgery	numbers and unit	
	- Number of clinical appointments		
	- Number of appointments with the surgeon		Face-to-face appointments, not group sessions
	- Duration of the first appointments with the surgeon		
	- Number of the appointments with the nutritionists		Incl. duration of appointments
	- Point in time of the decision regarding the surgical procedure	context	First/second/last appointment etc.
	- Reimbursement of surgical costs from the insurance		
	- Decision for the surgical procedure		Point in time, participation y/n
	- information provision approach/form of provided information		Group sessions, folder/map, one-on-one conversation with surgeon/nutritionist/coordinator

	- Groups session content		Profession of Groups session leader, number of group sessions, time of group sessions, group session content
	- Support group content		Support group content, cooperation with the support group y/n, support group is visited by a surgeon y/n, general information about the group (size, pre/post/both)
	- Additional information sources		Internet, social media, books etc.
	- FAQs pre-OP		
Pre-OP information	- Pre-OP general dietary information	context	
	- Pre-OP information about post-OP diet		
	- Pre-OP dietary supplements		Incl. costs
	- Pre-OP information about medication		
	- Pre-OP risk disclosure		
	- Pre-OP different surgical procedures		
	- Pre-OP pros and cons of the surgery		
	- Pre-OP drinking behavior		Separation of meals and drinking, sparkling beverages (carbonic acid)
Costs	- Costs for nutrition counseling	context	All information on costs regarding nutrition counselling
	- Other preoperative costs		
	- Other postoperative costs		
	- Reimbursement of nutrition counseling costs from the insurance pre-OP		
	- Reimbursement of nutrition counseling costs from the insurance post-OP		
Post-OP care/information	- Duration of follow-up care<br>- follow-up interval<br>- changes in eating behavior after surgery<br>- changes in everyday life after surgery<br>- FAQ post-OP	context	
Post-OP problems	- Dumping<br>- Malnutrition<br>- Management of small portions	context	

	- Reasons for post-OP problems		
Information needs	- judgement on information needs	context	
General problems	- Cooperation with general practitioners	context	
	- Responsibility of post-op blood screenings		
	- Psychotherapy		Need for pre/post-OP psychotherapy, costs and availability of psychotherapy, cooperation with psychotherapists
	- Patients emotions and fears		
Solutions	- Problem solutions	context	
