# Supplementary material for: Healthcare delivery and information provision in bariatric surgery in Germany: qualitative interviews with bariatric surgeons
Source: BMC Health Serv Res. 2021 Jul 5;21:659. doi: 10.1186/s12913-021-06629-4 (PMC8258934; doi:10.1186/s12913-021-06629-4)
Supplement: Supplementary file 3 — Additional file 3. [file 12913_2021_6629_MOESM3_ESM.pdf]

Supplement 3: Characteristics of the clinics

participant	surgical procedure	frequent surgical procedures	number of bariatric surgeries each year	number of bariatric surgeons at the clinic
B01	Bypass, Sleeve, GB	80% Bypass, 20% Sleeve	200	3
B02	Bypass, Sleeve, GB	60% Sleeve, 40% Bypass	100	3
B03	Bypass, Sleeve, GB	50 % Sleeve, 35 % Bypass	150	4
B04	Sleeve, Bypass, revision, conversion	80 % Sleeve, 10% Bypass, 10% revision	150	3
B05	Bypass, Sleeve, revision, conversion	70 % Bypass, 15 % Sleeve, 15 % conversion	500	4
B06	Bypass, Sleeve, revision, conversion, BPD	67% Bypass, 33% Sleeve	150	3
B07	Bypass, Sleeve, gastric balloon, BPD, gastric balloon, reversals	Sleeve	180	5
B08	Bypass, Sleeve, Revision	Bypass	200	2
B09	Bypass, Sleeve,	Sleeve	200	5
B10	Bypass, Sleeve, GB	50 % Bypass, 50% Sleeve	85	3
B11	Bypass, Sleeve, GB removal, revision, gastric balloon	Sleeve	100	5
B12	Bypass, Sleeve, revision, conversion, gastric balloon, GB removal	Bypass	70	2
B13	Bypass, Sleeve, GB, revision	Sleeve	100	2
B14	Bypass, Sleeve, GB, gastric balloon, Switch	Bypass, Sleeve	300	3
B15	Bypass, Sleeve, GB, gastric balloon	Sleeve	500	5

GB: gastric banding,
Bypass: referring to all kinds of gastric bypasses
